# Enhanced Performance of Inverted Perovskite Quantum Dot Light-Emitting Diode Using Electron Suppression Layer and Surface Morphology Control

**DOI:** 10.3390/ma16227171

**Published:** 2023-11-15

**Authors:** Hee Jung Kwak, Collins Kiguye, Minsik Gong, Jun Hong Park, Gi-Hwan Kim, Jun Young Kim

**Affiliations:** 1 Department of Semiconductor Engineering, Gyeongsang National University, Jinjudae-ro 501 beon-gil, Jinju-si 52828, Republic of Korea; kwakhj@gnu.ac.kr (H.J.K.); kiguye@gnu.ac.kr (C.K.); 2 Department of Materials Engineering and Convergence Technology, Gyeongsang National University, Jinjudae-ro 501 beon-gil, Jinju-si 52828, Republic of Korea; alstlr7973@naver.com (M.G.); yakte@gnu.ac.kr (J.H.P.); ghkim@gnu.ac.kr (G.-H.K.)

**Keywords:** perovskite, PeQDs, PeQLEDs, electroluminescence, light-emitting diodes, quantum dots, luminance, EQE, current density

## Abstract

The energy level offset at inorganic layer–organic layer interfaces and the mismatch of hole/electron mobilities of the individual layers greatly limit the establishment of balanced charge carrier injection inside the emissive layer of halide perovskite light-emitting diodes (PeQLEDs). In contrast with other types of light-emitting devices, namely OLEDs and QLEDs, various techniques such as inserting an electron suppression layer between the emissive and electron transport layer have been employed as a means of establishing charge carrier injection into their respective emissive layers. Hence, in this study, we report the use of a thin layer of Poly(4-vinylpyridine) (PVPy) (an electron suppression material) placed between the emissive and electron transport layer of a halide PeQLEDs fabricated with an inverted configuration. With ZnO as the electron transport material, devices fabricated with a thin PVPy interlayer between the ZnO ETL and CsPbBr_3_ -based green QDs emissive layer yielded a 4.5-fold increase in the maximum observed luminance and about a 10-fold increase in external quantum efficiency (EQE) when compared to ones fabricated without PVPy. Furthermore, the concentration and coating process conditions of CsPbBr_3_ QDs were altered to produce various thicknesses and film properties which resulted in improved EQE values for devices fabricated with QDs thin films of lower surface root-mean-square (RMS) values. These results show that inhibiting the excessive injection of electrons and adjusting QDs layer thickness in perovskite-inverted QLEDs is an effective way to improve device luminescence and efficiency, thereby improving the carrier injection balance.

## 1. Introduction

Since they were first reported [1], halide perovskite quantum dots (PeQDs) have gained serious research attention as viable candidates for use in next-generation lighting, large-area display technologies, solar cells, and lasers [2], owing to their high photoluminescence quantum yield (PLQY), tunable emissions wavelengths, and low-cost solution processing [3,4,5]. Recently, perovskite quantum dots (PeQDs) have been embraced for use as the emission layer (EML) material for high-performance displays because of their ability to produce 95% high over-threshold photoluminescence quantum yields (PLQY) and achieve high color purity with narrow full width at half maximum (FWHM) of 20 nm [6]. Halide perovskite QDs (CsPbBr_x_, X = Cl, Br, and I) have been proven to have practical applications owing to their high thermal stability and lower moisture sensitivity achieved through modulating their halide composition and quantum dot size. Despite halide perovskite QDs offering advantages of high efficiencies, low processing temperatures, cost-effectiveness, scalable fabrication, and short decay times similar to that of the intrinsic state of the art semiconductor materials, they are extremely sensitive to oxygen and moisture which limits their all-out commercialization. On closer inspection, CsPbX_3_ (X = Cl, Br, I) QDs have emerged as a sustainable option amongst all the available metal halide perovskite QDs as they are less susceptible to oxygen and moisture degradation than their counterparts [7]. Though CsPbX_3_ QDs have shown overall excellent performance characteristics, it is also important to select efficient hole transport and electron transport layer materials (HTL and ETL, respectively). Like in the case of OLEDs and QLEDs, Zinc Oxide (ZnO) is a promising candidate as an electron transport material for PeQLEDs as it has been proven to portray excellent electronic properties such as having high transparency, suitable energy band structure, and high electron mobility [8]. TFB (Poly[(9,9-dioctylfluorenyl-2,7-diyl)-co-(4,4′-(N-(4-sec-butylphenyl)diphenylamine)]) has registered significant success in studies on QLEDs, especially with Cd-based quantum dots, and hence stands out as the best HTL option to study the charge balance behavior in our PeQLEDs. Furthermore, TFB exhibits faster hole mobility (~1 × 10^−3^ cm^2^ v^−1^ s^−1^) when compared to many other organic HTLs, a high lowest unoccupied molecular orbital (LUMO) of 2.3 eV, and the ability to effectively block electron leakage which further makes TFB a suitable candidate for use as the hole transport material in our study [9].

To manufacture QLEDs with good device performance, establishing the charge injection balance is an important factor as it directly affects device lifetime and efficiency [10]. Normally, charge injection imbalance is caused by the accumulation of excess electrons inside the emissive layer stemming from the injection rate of electrons being faster than that of holes and also the rapid depletion of holes in the radiative recombination process, leading to build-up of excess electrons inside the emissive layer. This causes leakage of electrons into HTL as well as non-radiative Auger recombination [11]. This in turn causes serious degradation of the HTL which leads to a reduction in a device’s operational lifetime. Thus, it is important to regulate the rate at which electrons are injected into the emissive layer which will in turn reduce the rate of accumulation of excess charge carriers inside the emissive layer, leading to improved device performance.

Over the past few decades, to overcome problems caused by the accumulation of excess charge carriers inside the emissive layer, the structure of QLEDs has been designed using a variety of materials, including inorganic metal oxides, polymer, and small molecules as the transport layer materials [12]. In many studies, ZnO is utilized as the electron transport layer (ETL) in inverted QLED devices due to its high electron mobility, good carrier injection from ETL to QDs EML, and good energy level alignment [13,14,15,16].

Most laboratory-scale QLED devices are mainly constructed with ETL in direct contact with QDs EML. This contact results in exciton quenching and leakage current. Furthermore, the high carrier mobility of ZnO which is normally more than twice as high as that of the adjacent organic hole transport layer (HTL) results in more electrons being injected into the emissive layer than holes hence leading to carrier injection imbalance. This further outlines the importance of delaying electron entry into the emissive layer from ZnO ETL which aids in establishing carrier injection balance. For OLEDs and QLEDs, inserting an electron suppression interlayer between QDs EML and ETL has been employed as a means of delaying electron injection into the emissive layer. This has been proven to be a very effective way to improve charge carrier injection and the overall performance of QLEDs in various previous research [17,18]. This can also further prevent quenching by restricting the contact between the QDs EML and ETL, while slowing down the carrier injection rate from the ZnO ETL. Hence, in this work, we utilized a non-conjugate polymer, Poly(4-vinylpyridine) (PVPy) placed between the ZnO ETL and CsPbBr_3_ QDs EML, to study the effect of its presence on the overall performance of the PeQLEDs. PVPy has previously been reported to effectively delay electron injection into the emissive layer [17,19]. We also compared the surface morphology and electrical properties of ZnO deposited on bare glass and ZnO/PVPy films utilizing images obtained from AFM imaging and further improved the surface properties of the QDs layer by adjusting the concentration and spin speed of the spin-coating process. We studied the difference in the degree of dispersion resulting from using different concentrations of QDs solutions yielding QDs thin films with different thicknesses and different surface properties. Lower spin speeds produced thicker films which we in turn used to fabricate PeQLED devices. We characterized the device performance of the different devices fabricated with different QDs thicknesses to determine the optimal thickness for device fabrication.

## 2. Materials and Methods

### 2.1. Materials

Indium tin oxide (ITO glass substrates) used throughout the experiment were utilized without any surface modification. Cesium carbonate (Cs_2_CO_3_) (99%) was purchased from Sigma Aldrich (St. Louis, MI, USA). Tetraoctylammonium bromide (TOAB) (99%) was purchased from 1-materials (Dorval, QC, Canada). Didodecyldimethylammonium bromide (DDAB) (98%) and Octanoic acid (OTAc) (99%) were purchased from Macklin Inc. (Rochelle, IL, USA). Poly(4-vinylpyridine) (PVPy). Isopropyl alcohol(2-propanol) (IPA) and were purchased from Sigma Aldrich. Poly[(9,9-dioctylfluorenyl-2,7-diyl)-co-(4,4′-(N-(4-sec-butylphenyl)diphenylamine)] (TFB) and Molybdenum Oxide (MoO_3_) were purchased from OSM (Seoul, Republic of Korea). Silver (Ag) was purchased from TCI (Tokyo, Japan). Toluene, P-xylene, acetone, and ethyl acetate were purchased in Sigma Aldrich and received in the purest of forms and thus used without further purification. Material dilution was carried out only when required.

### 2.2. Synthesis of CsPbBr_3_ Quantum Dots and ZnO

The CsPbBr_3_ quantum dots were synthesized using a previously reported method with slight modifications [20]. First, 1 mmol of Cs_2_CO_3_ was added to 10 mL of OTAc in a vial and stirred at room temperature for 10 min. The PbBr_2_ precursor solution was then prepared by dissolving 1 mmol of PbBr_2_ and 2 mmol of TOAB in 10 mL of toluene. For CsPbBr_3_ QD synthesis, 1 mL of the cesium precursor solution was quickly dropped into 9 mL of a PbBr_2_-toluene solution in a vial. The reacting solution was stirred continuously for 5 min at room temperature in open air. Afterward, 3 mL of DDAB (in toluene 10 mg/mL) solution was added. Then, 2 min later, ethyl acetate was added to the solution in a volume ratio of 2:1, and after centrifugation, the remnant was collected and dispersed in toluene. The extra ethyl acetate was added to the toluene dispersion, and the precipitate was collected and re-dispersed in hexane.

The sol-gel ZnO nanoparticles were synthesized as follows: Samples of 0.316 g of zinc acetate, and 10 mL of ethanol were added to a flask and heated at 50 °C forming a turbid solution. Then 0.157 mL of ethanolamine was added to the reaction flask drop-wise until the solution in the reaction flask became transparent. The resulting mixture was stirred for an added duration of 120 min and later stored safely for experimental use.

### 2.3. PeQLED Device Fabrication

All devices were fabricated on glass substrates pre-coated with indium tin oxide (ITO). The size of the ITO-coated substrate is 25 mm × 25 mm. The substrates were cleaned through ultra-sonication with acetone (for 15 min) and isopropyl alcohol (IPA) (for 15 min) and dried for 4 h inside an oven set at 100 °C. The cleaned ITO glass substrates were treated with UV-ozone plasma for 15 min and then transferred into a nitrogen gas-filled glove box. The sol-gel ZnO solution that forms the ZnO NPs layer was spin-coated onto the ITO glass substrates at a spin speed of 3000 rpm for a duration of 40 s and annealed at 150 °C for 30 min to form the ZnO ETL layer. After, PVPy (0.3 mg/mL IPA) that forms the PVPy layer was then spin-coated at a spin speed of 3500 rpm for a duration of 45 s and then baked at 120 °C for 10 min. CsPbBr_3_ QDs (dispersed in hexane) were spin-coated at a spin speed of 3500 rpm for 45 s and then annealed at 60 °C for 10 min. TFB (20 mg/mL P-Xylene) was spin-coated onto the ITO glass substrates at 3500 rpm for 45 s and baked at 120 °C for 10 min. PVPy and TFB annealed for 10 min at 120 °C. The substrates were properly packaged and transferred into a thermal evaporator. Inside the thermal evaporator, MoO_3_ (10 nm) and Ag (80 nm) were deposited -onto the substrates sequentially through a shadow mask under high vacuum conditions of ~4 × 10^−6^ torr. MoO_3_ was deposited at a rate of 0.3 Å/s and Ag was deposited at a rate of 1.0 Å/s. After thermal deposition, the devices were encapsulated with ultraviolet-curable resin and encapsulation glass inside the glove box. The device’s active area was 2.25 mm^2^.

### 2.4. PeQLED Device Characterization

The performance characteristics of the PeQLED devices were determined using a Keithley 2400 source and a PR-655 Spectra Scan spectrophotometer. For the surface properties of CsPbBr_3_ QD properties, TEM images were obtained using the TF30ST (FEI) (Hillsboro, OR, USA), and XRD characteristic imaging was carried out using the D8 Advance A25 Plus (Bruker) (Billerica, MA, USA). The surface morphology of the CsPbBr_3_ QD layer and surface characteristics were obtained through XE-100 (Park Systems) (Suwon, Republic of Korea) atomic force microscopy imaging.

## 3. Results and Discussion

### 3.1. Characterization of CsPbBr_3_ QDs

**Figure 1:**
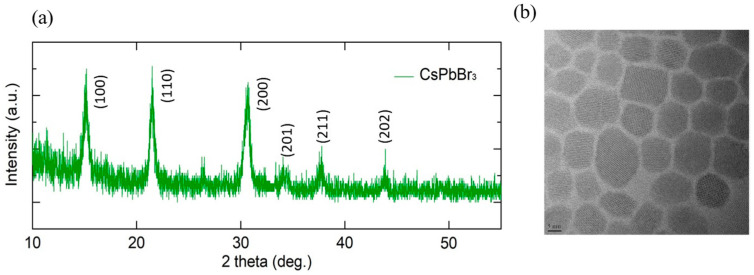
(**a**) XRD pattern plot and (**b**) TEM images of CsPbBr_3_ QDs. The average diameter of CsPbBr_3_ is about 10 nm.

Figure 1a shows the Intensity-2theta XRD pattern plot of the data on CsPbBr_3_ QDs obtained from XRD imaging. The plot provides details on the crystallinity of CsPbBr_3_ QDs. In addition, Figure 1b is a Transmission Electron Microscope (TEM) image of the CsPbBr_3_ QDs. The crystals were confirmed to have an average diameter of about 10 nm.

### 3.2. Performance of Perovskite Inverted QLEDs

**Figure 2:**
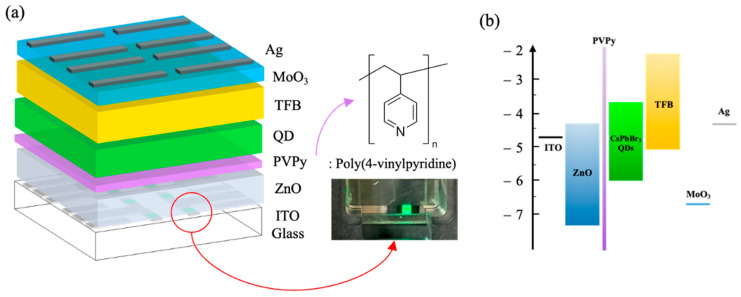
(**a**) Schematic diagram of perovskite inverted QLEDs with a thin PVPy interlayer. Inset, the chemical structure of PVPy with a picture of the fabricated device emitting green light during testing. (**b**) Energy level diagram of PeQLED devices fabricated with PVPy.

A schematic diagram of the perovskite QLEDs fabricated with a PVPy interlayer used throughout this work is shown in Figure 2a. The devices were fabricated with an inverted configuration of ITO/sol-gel ZnO (35 nm)/PVPy(0.3 mg/mL IPA)/CsPbBr_3_ QDs/TFB(50 nm)/MoO_3_(10 nm)/Ag(100 nm). Between ZnO ETL and CsPbBr_3_ QDs, a thin layer of PVPy was inserted to serve as an electron suppression (blocking) layer, hence regulating the rate of electron injection into the CsPbBr_3_ QD emissive layer. Inset, the chemical structure of PVPy and a picture showing green light emission from one of the fabricated PeQLED devices are attached to Figure 2a. Figure 2b depicts the energy level diagram of the PeQLED devices fabricated with PVPy.

Initially, we fabricated PeQLED devices without a PVPy interlayer so as to assess the effect of the presence of the PVPy interlayer on the overall performance of the PeQLED device. The respective devices (ones without PVPy and ones with a PVPy interlayer) were assessed in regard to the overall performance characteristics.

**Figure 3:**
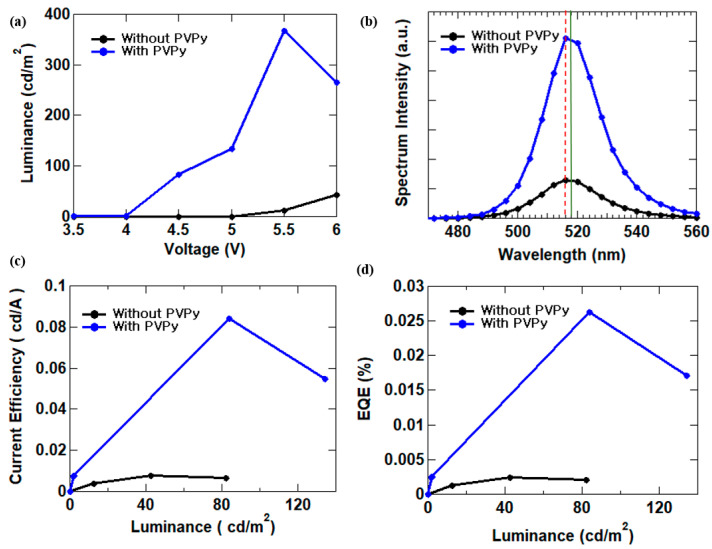
I-V-L measurement of QLEDs (**a**) luminance–voltage; (**b**) spectrum intensity; (**c**) current efficiency–luminance; (**d**) EQE–luminance.

The luminance-voltage plots are shown in Figure 3a of a device fabricated with PVPy to one without PVPy. A 4.5-fold increase in maximum observed luminance from 82.03 cd/m^2^ to 367.9 cd/m^2^ in devices manufactured with PVPy and a reduction in the turn-on voltage were found. This implies that the insertion of PVPy improved the charge imbalance. In Figure 3b, devices with PVPy have a much stronger EL intensity in comparison to those fabricated without PVPy at the same voltage. Further, the electroluminescence (EL) peak of the devices fabricated with PVPy was noticed to have a slightly shifted from about 516 nm (represented by the red dotted line in Figure 3b) to 518 nm (represented by the green line in Figure 3b) and Table 1. This is caused by the change in distance between the cathode and to anode due to the insert of the PVPy layer and shift in the exciton recombination zone [21]. We also noticed that with the addition of the PVPy electron suppression layer, the current efficiency increased by about 11-fold and the external quantum efficiency (EQE) increased by about 8.6-fold when compared with devices without PVPy as shown in Figure 3c,d and Table 1. This is a result of the PVPy electron suppression layer acting as a barrier slowing down the rate of electron injection into the emissive layer, thereby also reducing the rate of excessive electron build-up inside the emissive layer hence promoting efficient recombination inside the emissive layer [17,19]. This thus localizes the hole-electron recombination to occurring more inside the emission layer than inside any other functional layer, which greatly improves the performance of the device.

### 3.3. CsPbBr_3_ QD Morphology Control

**Figure 4:**
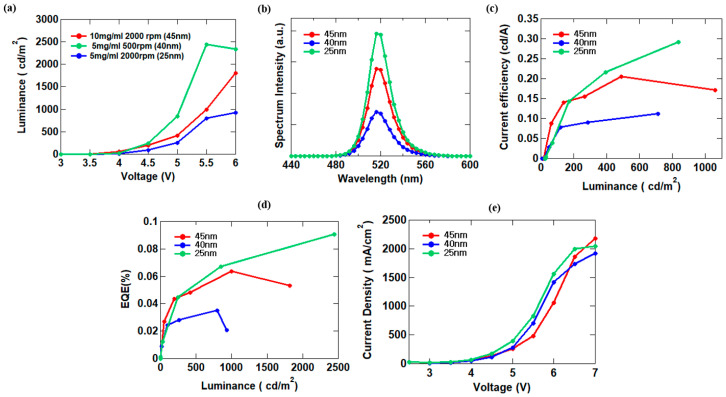
J-V-L measurement of QLEDs with different QD thicknesses: (**a**) luminance–voltage; (**b**) spectrum intensity; (**c**) current efficiency–luminance; (**d**) EQE–luminance; and (**e**) current density–voltage.

Thus far, we confirmed through experiments that the presence of the PVPy serving as an electron suppression layer effectively improves the device efficiency of PeQLED devices. Furthermore, we studied the effect of changing the surface characteristics and different layer thicknesses of the CsPbBr_3_ QDs layer on the overall efficiency of the devices. We prepared 5 mg/mL and 10 mg/mL concentrations of CsPbBr_3_ QDs in hexane which we deposited onto thin glass substrates at a rotation speed of 2000 rpm. The thickness of each film was determined through atomic force microscopy (AFM) imaging and the results were 25 nm for the 5 mg/mL QDs solution and 45 nm for the 10 mg/mL QDs solution. For relative comparison, we also determined the thickness of the film formed by spin-coating a 5 mg/mL QDs solution at a lower spin speed of 500 rpm which yielded a thickness of 40 nm. All thin films were annealed at 60 °C for 10 min. After determining the thicknesses of the films formed by the different QDs solutions, we used these QDs solutions and their respective spin conditions to fabricate different PeQLED devices with inverted device configurations. Figure 4 shows the performance metrics of the fabricated devices. In Figure 4a, 5 mg/mL QDs spin-coated at 500 rpm spin speed (40 nm) represented by the green characteristic line showed the highest luminance of 2493 cd/m^2^. The 10 mg/mL QDs that yielded a thickness of 45 nm represented by the red characteristic line had a lower peak luminance of 1082 cd/m^2^. The 5 mg/mL QDs solution spin-coated at 2000 rpm (25 nm) showed an even lower peak luminance of 927.2 cd/m^2^. As shown in Figure 4c,d, the device fabricated with 5 mg/mL QDs solution deposited at 500 rpm spin speed yielded the best device performance with a current efficiency of 0.29 cd/A and EQE of 0.09%. This result can be attributed to the thickness formed by the 5 mg/mL concentration QDs solution and the spin-speed being the optimum thickness for the devices as it was found that either decreasing or increasing the thickness produced reduced current efficiency and EQE values. To explain, the efficiency was observed to be higher when the QDs layer thickness was 45 nm, but not as high as when the thickness was 40 nm. Also, the efficiency significantly decreased when the QDs layer thickness was 25 nm. Current efficiency decreased following a similar order. That is 0.2 cd/A for a 45 nm thick QDs layer then 0.1 cd/A for a 25 nm QD layer. The EQE decreased in a similar trend that is 0.06% for the 45 nm QDs layer to 0.03% when the QDs thickness was 25 nm. The results are shown in Table 2. With these results, we firmly establish that the 40 nm-thick QDs layer was the most appropriate for use in the experiment. We confirmed that the thickness of the QDs layer has a significant impact on the performance of the PeQLEDs in that when the QDs layer is too thin, there is carrier leakage into the emissive layer due to the spherical properties of the quantum dots resulting in low efficiency. A gradual increase in the thickness of the QDs layer initially suppresses carrier leakage leading to higher efficiency. However, continuous increase in thickness affects the PeQLED device due to the slower transport properties of the thicker QDs films resulting in reduced maximum observed luminance and EQE values along with a decrease in current density (Figure 4e) [22].

**Figure 5:**
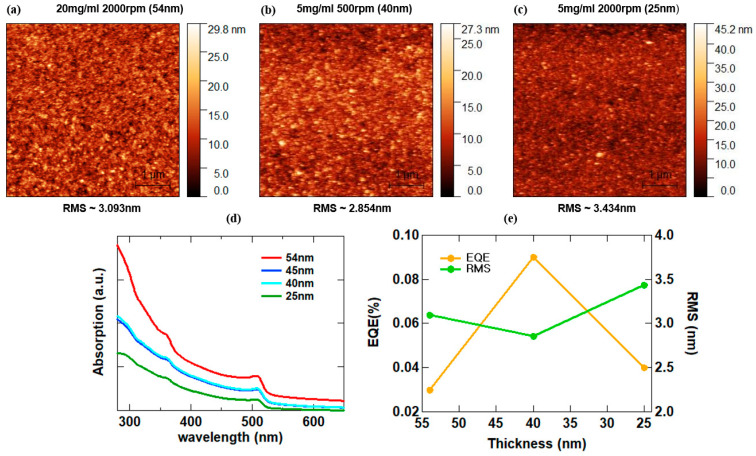
AFM images of CsPbBr_3_ QD layer depending on thickness. (**a**) 54 nm, (**b**) 34 nm, (**c**) 25 nm, and graph of each layer properties. (**d**) UV-Vis absorption of each QD layer (**e**) EQE-RMS-Thickness.

We utilized AFM to identify the surface properties of the CsPbBr_3_ QDs layer according to the thickness and thin film quality. Figure 5a–c show surface images of 54 nm, 40 nm, and 25 nm thin films deposited onto bare glass and their corresponding RMS values. The 40 nm QDs films that yielded the highest device efficiency had the least RMS value, 2.854 nm. The thicker 54 nm thin film had an RMS value of 3.093 nm, and the thinner 25 nm thin film had an RMS value of 3.434 nm. The RMS values and their respective EQE are compared in Table 3. The devices fabricated with 54 nm thin film yielded an EQE of 0.03%, while one fabricated with 25 nm thin film yielded an EQE of 0.04%. Devices using a 40 nm thin film yielded the lowest RMS value and recorded the highest EQE of 0.09%. Figure 5d shows a UV-visible absorption graph. The intensity of the absorption peak varied in line with the QDs layer thickness, and the thicker the QDs thin film, the more absorption occurs. That is a change in thickness of the QDs EML exhibited different optical characteristics along with the surface morphology. The observed peaks might possibly occur as a result of the presence of Pb traces [23]. A detailed summary of the results of the experiment, the RMS values of the different thin films formed by the QDs obtained from AFM imaging were compared to their corresponding EQE values as shown in Figure 5e and Table 3. From this comparison, we deduced that the change in morphology characteristics brought about by the changing QDs thickness improved the luminance and device efficiency. The thin films with lower RMS values yielded great overall results with the best results obtained when the QDs thickness is 40 nm. At 40 nm QDs thickness, the RMS value of the thin film reduced implying improvement in the surface properties. These results demonstrate that optimizing the thickness and surface properties of the QDs emissive layer needs to be put into serious consideration. 

## 4. Conclusions

In summary, we utilized a thin electron suppression layer of Poly(4-vinylpyridine) (PVPy) as an interlayer between sol-gel ZnO used as the electron transport layer (ETL) and CsPbBr_3_ QDs layer used as the emissive layer to establish the charge injection balance inside the QDs emissive layer from the electron transport layer and further improve the device efficiency in inverted PeQLEDs. In addition, the thickness of the CsPbBr_3_ QDs with appropriate surface properties was determined by adjusting the concentration of QDs and using different spin conditions for the spin-coating process. It was determined that a 40 nm-thick QDs layer was optimal for use in the PeQLED device fabrication. Using a 40 nm-thick QDs EML layer yielded a peak luminance of 2439 cd/m^2^ and an EQE of 0.09%. Increasing the QDs emissive layer thickness (using QDs thickness > 40 nm) produced a drop in the device performance particularly in the EQE characteristic. (QDs thickness: 54 nm, EQE: 0.03%). Also using a thinner QDs layer thickness (QDs thickness > 40 nm) produced decreased EQE values (QDs thickness: 25 nm, EQE: 0.04%). On overall inspection, a 30-fold enhancement in device efficiency was observed with devices fabricated with a PVPy electron suppression layer as compared to those fabricated without an electron suppression layer. We present a method to easily improve charge imbalance by utilizing PVPy layers in PeQLED. In addition, we further propose a way to optimize the thickness of the QDs layer and surface properties of the emission layer by adjusting the concentration of QDs and the speed of spin-coating to obtain the best possible device performance metrics.

## Figures and Tables

**Table 1:** Performance of perovskite inverted QLEDs with and without PVPy layer.

Device	EL λ_max_ [nm]	Max. Luminance [cd/m^2^]	Max. Current Efficiency [cd/A]	Max. EQE [%]
Without PVPy	516	82.03	0.0076	0.003
With PVPy interlayer	518	367.9	0.084	0.026

**Table 2:** Performance of perovskite-inverted QLEDs with different thicknesses of CsPbBr_3_ QDs.

	EL λ_max_ [nm]	Max. Luminance [cd/m^2^]	Max. Current Efficiency [cd/A]	Max. EQE [%]
10 mg/mL 2000 rpm (45 nm)	518	1082	0.204	0.064
5 mg/mL 500 rpm (40 nm)	518	2439	0.29	0.091
5 mg/mL 2000 rpm (25 nm)	516	927.2	0.1123	0.035

**Table 3:** EQE and RMS values according to CsPbBr_3_ thickness.

Thickness [nm]	EQE [%]	RMS [nm]
54	0.03	3.093
40	0.09	2.854
25	0.04	3.434

## Data Availability

Data will be made available on request.
